# Conserved versatile master regulators in signalling pathways in response to stress in plants

**DOI:** 10.1093/aobpla/plt033

**Published:** 2013-08-01

**Authors:** Victor E. Balderas-Hernández, Miguel Alvarado-Rodríguez, Saúl Fraire-Velázquez

**Affiliations:** 1 Laboratorio de Biología Integrativa de Plantas y Microorganismos, Unidad Académica de Ciencias Biológicas, Universidad Autónoma de Zacatecas, Av. Preparatoria s/n, Col. Agronómica, CP 98066, Zacatecas, México; 2 Laboratorio de Cultivo de Tejidos Vegetales, Unidad de Agronomía, Universidad Autónoma de Zacatecas, Carr. Zacatecas-Jerez km 17, CP 98000, Zacatecas, México

**Keywords:** Biotic/abiotic stress, co-activators, gene expression regulation, integrators, key regulators, plant stress response.

## Abstract

From the first land plants to the complex gymnosperms and angiosperms of today, environmental conditions have forced plants to develop molecular strategies to surpass natural obstacles to growth and proliferation, and these genetic gains have been transmitted to the following generations. In this long natural process, novel and elaborate mechanisms have evolved to enable plants to cope with environmental limitations. Elements in many signalling cascades enable plants to sense different, multiple and simultaneous ambient cues. A group of versatile master regulators of gene expression control plant responses to stressing conditions. For crop breeding purposes, the task is to determine how to activate these key regulators to enable accurate and optimal reactions to common stresses. In this review, we discuss how plants sense biotic and abiotic stresses, how and which master regulators are implied in the responses to these stresses, their evolution in the life kingdoms, and the domains in these proteins that interact with other factors to lead to a proper and efficient plant response.

## Abstract (precis)

Environmental conditions have forced plants to develop elaborated molecular strategies to surpass natural obstacles to growth and proliferation. Elements in multiple signaling cascades allow plants to sense multiple and simultaneous ambient cues, and establish an opportune defensive response. A group of versatile master regulators of gene expression are decisive to control plant responses to stressing conditions. For crop breeding purposes, the task is to determine how to activate these key regulators to enable accurate and optimal responses to stressing conditions. In this review, we discuss how and which master regulators are implied in the responses to biotic and stresses, their evolution in the life kingdoms, and the interaction with other molecular factors that lead to a proper and efficient plant response.

## Introduction

Plants are continuously exposed to harmful environmental conditions, and biotic and abiotic stressors limit crop yield and also the land-use on earth. To guarantee success in the adaptation and survival to limiting growth conditions, plants have developed diverse stress-responsive signalling pathways. Once adverse environmental cues are perceived, they are transmitted to different cellular action centres, resulting in activation of mechanisms that prepare the plant for adaptation. The expeditious integration of the stress signals and the activated adaptation/defence mechanisms allow plants to grow in adverse environments. Plant stress evasive strategies involve a multilevel reorganization with changes in energetic, metabolic, transcriptional, growth and proliferation profiles. This massive and complex restructuring is dynamically regulated in response to the type, severity and duration of one or a combination of stresses (Atkinson and Urwin 2012). Plants are able to display strategic defence responses when two stressors occur at the same time, and this response can be, in some cases, distinctive from the response to either individual stress (Koussevitzky *et al.* 2008). Bipartite protective responses may indicate that plants economize molecular resources in order to improve the chances of survival. These differential responses also provide evidence for molecular components that coordinately integrate multiple signals and responses such as extensive gene expression reprogramming (Munoz and Castellano 2012). These master regulators positively or negatively control the transcription of a wide variety of genes that are involved in the mechanisms for plant adaptation and survival. These central regulatory hubs allow for a rapid and efficient transcriptional remodelling, increasing the plasticity in the general stress response. Master regulators may directly associate with the promoter regions of genes or may indirectly control gene expression by activation of transcription factors (TFs) or general repressors. Some master regulators are able to directly inhibit the activity of key metabolic enzymes that are decisive for energy homoeostasis in the cell. Some of the most important master regulators found in plants have a high degree of cross-species conservation. This evolutionary conservation is observed at both structural and functional levels.

In this work, we review the current state of our knowledge of master regulators of transcription in plants involved in the response to environmental constraints. We discuss their key roles in plant adaptation during adverse conditions of biotic and abiotic stresses. We analyse their regulatory activities, their dynamic and specific conformation, their interaction with associated molecules, type-stress specificity, possible participation in different stress-signalling pathways and their evolution among life kingdoms. Finally, we discuss the relevance of these master regulators to engineering of crops to meet the needs of the changing world.

## Master Regulators of Signalling Cascades that Respond to Biotic Stress

The plant defence response to pathogens involves the perception of pathogen-associated molecular patterns (PAMPs) by pattern recognition receptors and the activation of the basal immune response; this immunity response is called pattern-triggered immunity (PTI) (Jones and Dangl 2006; Lacombe *et al.* 2010). Some microbial pathogens possess effectors that counteract the function of components in the PTI signalling cascade. Plant disease resistance proteins may then induce a gene-for-gene resistance described by Flor (1971); this second level of plant defence response is known as effector-triggered immunity (ETI) (Abramovitch *et al.* 2003; Gassmann and Bhattacharjee 2012). Recent results suggest that pattern recognition receptors interact physically with resistance proteins, evidence that PTI and ETI receptors can reside in the same protein complex and that PTI and ETI signalling likely interact at very early stages (Qi *et al.* 2011). The increase in cytosolic Ca^2+^ is an early event in the elicitor-sensing mechanism in plant cells; calcium signatures contain encrypted information that is decoded into specific biological responses (Scrase-Field and Knight 2003; Lecourieux *et al.* 2006; Monshausen 2012). In the plant–pathogen interaction, plants often release peptide signals referred to as damaged-associated molecular patterns (DAMPs). These molecules also induce defence responses to the microbial intruders (Krol *et al.* 2010; Ma *et al.* 2012). The activation of the defence responses by PAMPs and DAMPs induces a cytosolic Ca^2+^ burst. Recent studies have linked cGMP-activated Ca^2+^-conducting ion channels to the induction of  immune response signalling. These receptors actuate synergistically to generate a Ca^2+^ signal signature that eventually results in defence gene expression and the hypersensitive response (Ma *et al.* 2012).

### Non-expressor of pathogenesis-related protein, an ankyrin repeat protein, a master regulator of the biotic stress response

**Figure 1.**
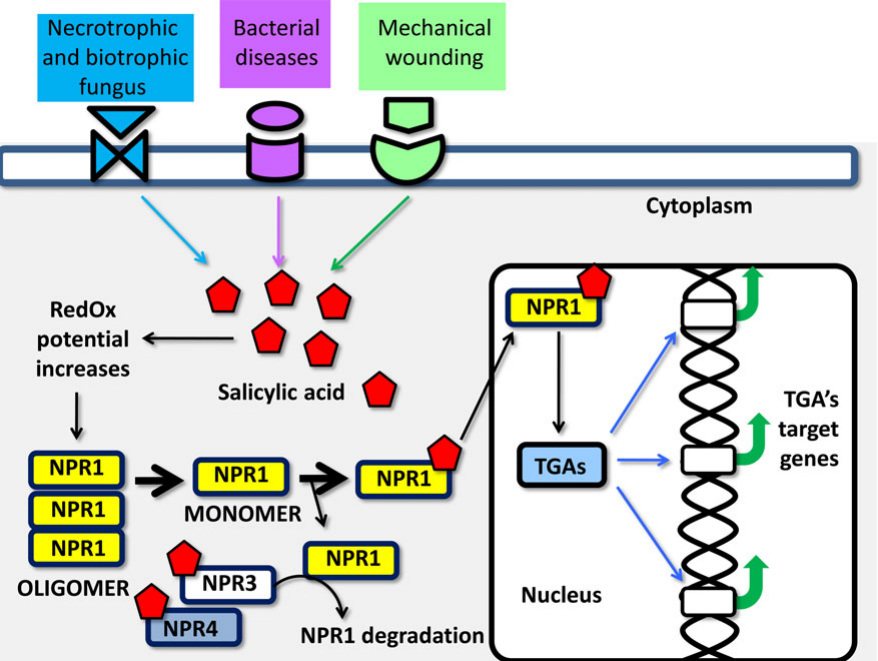
NPR1 is a master transcriptional regulator of genes activated by different biotic stresses. Diverse biotic stresses (attack by pathogenic bacteria and fungi) or mechanical stressors (herbivory or wounding) cause an increment in the intracellular levels of SA. This increment causes an elevation in the redox status of the cell that in consequence promotes the monomerization of NPR1 (*n*on-expressor of *p*athogenesis-*r*elated (PR) protein genes) oligomers. Then monomers of NPR1 act as receptors for SA and can be translocated to the nucleus to exert its regulatory activities. Once in the nucleus, NPR1 interacts with TGA2, TGA5 and TGA6 transcriptional factors. These interactions are essential to activate the transcription of PR genes and induce SAR. NPR3 and NPR4 will act also as SA receptors and promote NPR1 degradation via proteasome.

A first characteristic step in the induction of defence against pathogens in plants is an increase in the level of endogenous salicylic acid (SA); this increase changes the redox state in cells. In turn, this causes monomerization of *n*on-expressor of *p*athogenesis-*r*elated protein (NPR1) (Mou *et al.* 2003). NPR1 is an ankyrin repeat protein that was initially identified as a central regulator of the systemic acquired resistance (SAR) in *Arabidopsis thaliana* (Cao *et al.* 1994, 1997). NPR1 is involved in the regulation of the transcription of a number of pathogenesis-related (PR) genes (Pieterse *et al*. 1998; Zhang *et al*. 1999). Resistance to several necrotrophic and biotrophic fungi, to certain bacteria and to nematodes results from overexpression of exogenous or endogenous *NPR1* in various plant species with apparently minimal or no pleiotropic effects (Cao *et al.* 1998; Wally *et al.* 2009; Parkhi *et al.* 2010). In rice, the orthologue *OsNPR1* is up-regulated upon herbivore infestation or mechanical wounding (Li *et al*. 2012*b*). Based on these data, NPR1 is a positive regulator of the plant defence response to biotic stress and is considered a master regulator of the defence reaction (Fig. 1). In resting cells in plants, NPR1 is an oligomer localized to the cytoplasm, but under pathogen challenge, NPR1 oligomers dissociate into monomers. In the monomer, nuclear localization signals are exposed and NPR1 migrates to the nucleus; the nuclear localization of NPR1 is essential for inducing the transcription of PR genes and SAR activation (Kinkema *et al.* 2000). In the absence of pathogen, the low amounts of NPR1 that reach the nucleus are degraded by the proteasome, preventing its co-activator activity. To induce SAR, a large amount of NPR1 monomers must be translocated to the nucleus. In the nucleus, NPR1 interacts with specific TFs to initiate target gene transcription by recruiting the transcription initiation complex (IC) and RNA polymerase II (PolII). There is some evidence that NPR1 is phosphorylated by a kinase associated with the IC, and the phosphorylated NPR1 becomes a target for ubiquitinylation and degradation by the proteasome. Fresh NPR1 is required to reinitiate the transcription cycle, explaining the correlation between the rate of NPR1 degradation and the amplitude of target gene transcription (Spoel *et al.* 2009).

NPR1 is the receptor of the endogenous phytohormone SA (Wu *et al*. 2012). NPR3 and NPR4, two paralogues of NPR1, also bind SA with different affinities. NPR3 and NPR4 act as Cullin 3 (CUL3) ubiquitin ligase adaptors for NPR1 proteasome degradation. In the absence of pathogen challenge when the levels of SA are low, most NPR1 are removed by CUL3-NPR4-mediated degradation. Even at basal levels of SA, some amount of NPR1 escapes degradation (Fu *et al.* 2012). It is also possible that accumulation of H_2_O_2_ in cytoplasm prevents the nuclear translocation of NPR1 (Peleg-Grossman *et al.* 2010). In *Nicotiana tabacum*, NtTTG2, a protein bearing a WD40 protein interaction domain, impedes the nuclear localization of NPR1 abolishing PR gene transcription (Li *et al*. 2012*a*).

NPR1 is a negative regulator of signalling through another phytohormone, jasmonic acid (JA) (Spoel *et al.* 2003; Gfeller *et al.* 2010). In rice, the antisense expression of *OsNPR1* (as-npr1) results in a 50 % reduction in gene transcription and increased levels of JA, ethylene (ET) and herbivore-induced trypsin proteinase inhibitors. The antisense expression also reduced the effects of the rice striped stem borer (*Chilo suppressalis*) (Li *et al*. 2012*b*). The master regulator function in NPR1 is explained in part by a broad complex, tramtrack bric à brac/poxvirus and zinc finger (BTB/POZ) domain located in the N-terminal region, through which it interacts with TGACG motif-binding (TGA) bZIP-type TFs (TGA2, TGA5 and TGA6), and a C-terminal transactivation domain (Cys-oxidized domain) required for the specific interaction with TGA2 to form a transactivating complex called the enhanceosome (Rochon *et al.* 2006). PR gene expression in SAR is dependent on the functionally redundant TGAs (Zhang *et al*. 1999) (Fig. 1). TGA TFs were first described in pea and tobacco. These TFs recognize repeats of TGACG motifs in the 35S promoter of cauliflower mosaic virus, originally named activation sequence factor 1 (Lam *et al.* 1989). The TGA TFs in plants are involved in the expression of defence genes in response to SA (Lebel *et al.* 1998), and their interaction with NPR1 enhances their DNA binding activity (Despres *et al.* 2000). Other NPR1-interacting proteins include NIM1-interacting (NIMIN) proteins, NIMIN-1, NIMIN-2 and NIMIN-3. NIMIN-1 and -2 proteins interact with the C-terminal regions of NPR1 through a common binding motif, whereas NIMIN-3 interacts with the N-terminal region of NPR1. These NPR1–NIMIN heterodimers interact with the basic leucine zipper TFs of the TGA family (Weigel *et al.* 2001). There are some differences in sequences between NPR1 proteins from plant species, for example, between *A. thaliana* and *N. tabacum*, but NPR1 and NPR1-like proteins all harbour the penta-amino acid motif LENRV and a strictly conserved binding site for NIMIN proteins. It appears that distinct threshold levels of cellular SA are sensed by SA-sensitive complexes formed by NPR1 and NIMIN proteins (Maier *et al.* 2011).

**Figure 2.**
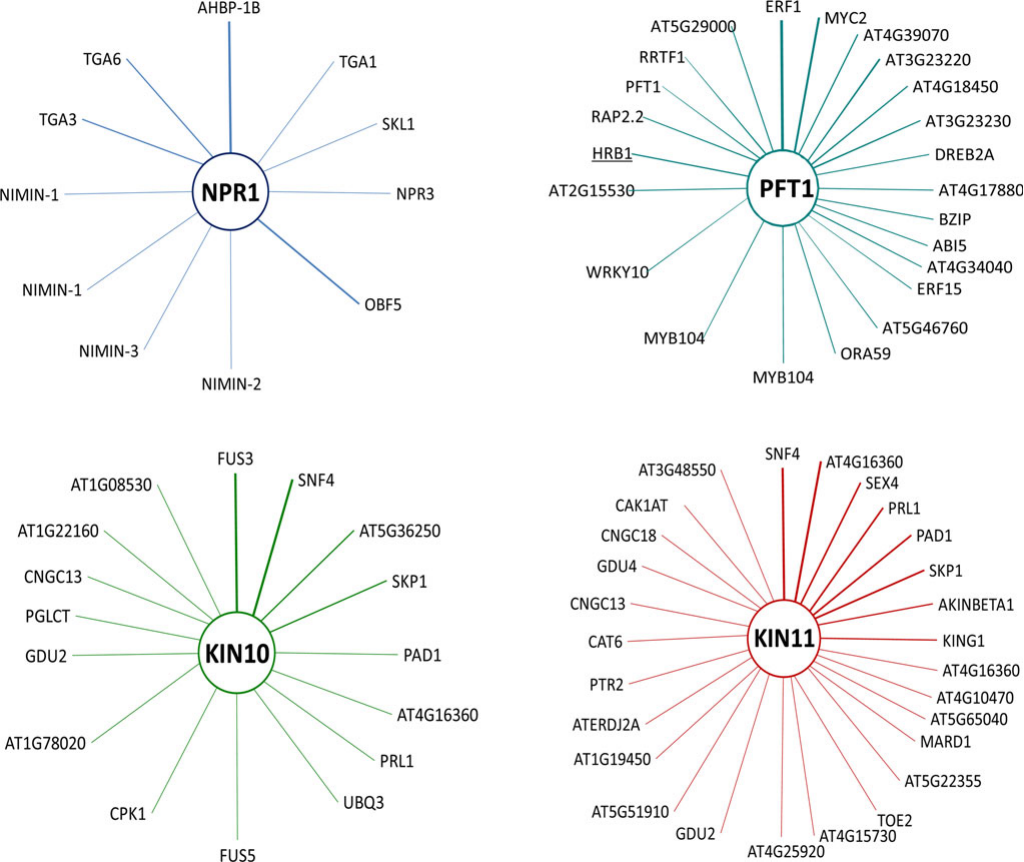
Interactome maps for NPR1, PFT1, KIN10 and KIN11. Maps of interaction for *A. thaliana* constructed with physical and genetic interaction data from the BioGRID database server. PFT1 (Med 25); KIN10 and KIN11 mean SnRK1 protein kinases. Main interactors with NPR1: TGA TFs and NIMIN1–3 proteins. Main interactors with PFT1: MYB, DREB, MYC and WRKY TFs. Main interactors with SnRK1 protein kinases: proteins of diverse biological functions. Underlaid interactors represent the genetic interactions. For more information on each interaction, refer to Tables 1–4.

The phytohormones JA and ET are involved in plant defence against herbivores and necrotrophic pathogens and are key signalling molecules in the induction of resistance. There is evidence that the phytohormone signalling pathways are interconnected (Pieterse *et al.* 1998; Asai *et al.* 2000; Clarke *et al.* 2000; Mhamdi *et al.* 2010). Generally, SA has an antagonistic effect on JA signalling. *Arabidopsis* plants with low levels of endogenous SA have higher levels of JA and enhanced expression of JA-induced genes in response to bacterial infection, whereas SA accumulation in wild plants upon pathogen infection suppresses JA signalling. Similarly, in studies of SA and JA exogenous application in plants, SA inhibits JA synthesis and signalling and JA-responsive gene expression by a mechanism of redox modulation (Koorneef *et al.* 2008). Studies in the *Arabidopsis* mutant *npr1*, which lacks the SA signalling cascade, demonstrated that NPR1 is a central regulator that controls the suppression of JA signalling. The crosstalk between these pathways is modulated by cytosolic NPR1 (Spoel *et al.* 2003). Ethylene modulates the role of NPR1 in the SA–JA pathways crosstalk (Leon-Reyes *et al.* 2009). The notable NPR1 participation as a central regulator in biotic stress response in plants is highlighted in a genome-wide gene expression and network analysis in *A. thaliana* inferred from an assembly of available microarray data, where the results show that this plant species has evolved regulatory networks and subnetworks with high connectivity in terms of transcriptional regulation in response to changing environments; in these subnetworks, in particular, in the SAR, 2 of the 12 nodes are NPR1 and NIMIN1, NPR1 furthermore reinforced with experimental reported data (Carrera *et al.* 2009). We inferred the interactome network for NPR1 (*At1g64280*) (BioGRID ID: 27954) using data available from *A. thaliana* with BioGRID version 3.2.99 available online (http://thebiogrid.org) (Stark *et al.* 2011)*.* The interactome network including physical and genetic interaction data excluding self-interactions contains a total of 35 interactions at low confidence level. The outstanding interactors are TGA TFs and NIMIN1–3 proteins (Fig. 2, Table 1).

**Table 1.** Transcription factors and proteins in interaction with NPR1 (At1g64280) in *A. thaliana* inferred from the BioGRID database.

Interactor	Gene ID	Short description
AHBP-1B	830586	Transcription factor TGA2
OBF5	830587	Transcription factor TGA5
TGA3	838812	Transcription factor TGA3
TGA6	820405	Transcription factor TGA6
NIMIN-1	837800	Protein NIM1-interacting 1
NIMIN-3	837464	Protein NIM1-interacting 3
NIMIN-2	822184	Protein NIM1-interacting 2
TGA1	836646	Transcription factor TGA1
NPR3	843221	NPR1-like protein 3
SKL1	822306	Shikimate kinase like 1

### Mediator complex, a sophisticated master regulator of the response to biotic stress

In the last two decades, several molecular players that integrate signals from signalling pathways activated in response to biotic stress have been described. One of these is the Mediator complex, the conserved four-module multiprotein unit initially discovered in yeast and later described in fungi, metazoans and plants (Mathur *et al.* 2011). Mediator is involved in the RNA PolII-catalysed transcription (Kelleher *et al.* 1990). It is an essential component in the transcriptional machinery in eukaryotes (Bourbon 2008) that promotes the assembly and activation of transcription complexes on core promoters, interacts with RNA PolII in the initiation of transcription and serves as a primary conduit of regulatory information from enhancers to promoters, integrating positive and negative regulatory information (Myers and Kornberg 2000; Kuras *et al.* 2003). Mediator is a multicomponent complex composed of at least 34 subunits in plants; 25 and 30 subunits are found in yeast and metazoans, respectively (Mathur *et al.* 2011). *Arabidopsis* Mediator subunits have very low homology compared with other species. In *Arabidopsis*, there are at least 21 conserved and six novel (specific) Mediator subunits (Backstrom *et al.* 2007). A Mediator subunit with an integrative signalling function characterized in *A. thaliana* and other plant species is Med25. In *A. thaliana*, the *PHYTOCHROME AND FLOWERING TIME1* (*PFT1*) gene encodes the Med25 subunit (Backstrom *et al.* 2007) and is required for jasmonate-dependent defence gene expression and basal resistance to leaf-infecting necrotrophic fungal pathogens, acting as a positive regulator of defence gene expression. Interestingly, *PFT1* is a susceptible host factor that facilitates the colonization by *Fusarium oxysporum*, a root-infecting hemibiotrophic fungal pathogen that requires intact JA-dependent signalling in the host (Kidd *et al*. 2009; Thatcher *et al*. 2009). Med25 in *Arabidopsis* regulates a spectrum of signalling pathways by means of selective interaction with specific TFs that differentially regulate the JA and abscisic acid (ABA) cascades. Med25 interacts physically with the MYC2 TF in the promoter regions of *MYC2* target genes to enhance their expression. MYC2 and Med25 also interact with ABA-Insensitive 5 (ABI5), a leucine zipper TF, in the promoter regions of *ABI5* target genes and have a negative effect in the expression of these genes (Chen *et al*. 2012*b*). Med25 interacts directly with three TFs of the AP2-EREBP (APETALA2 and ET-responsive element binding proteins) family, and these three TFs interact directly with the GCC-box of *PDF1.2*, a gene regulated also under the cascade of JA, suggesting that Med25 regulates *PDF1.2* transcription (Ou *et al.* 2011).

Furthermore, Med25 participates in regulating essential developmental processes, such as flowering and organ size determination, integrates environmental cues to development control (Elfving *et al.* 2011) and is involved in controlling root hair differentiation by maintaining reactive-oxygen species distribution (Sundaravelpandian *et al.* 2013). In the signalling to the process of flowering induction, two RING-H2 proteins target Med25 for degradation by a mechanism called ‘activation by destruction’. Proteolysis of Med25 is necessary for transcription of the *FLOWERING LOCUS T* gene (Inigo *et al.* 2012*a*). A study under contrasting conditions of temperature and light quality, transcriptome comparisons of *Arabidopsis pft1* (a *Med25* mutant) and the transcriptome after *F. oxysporum* attack found that *Med25* is at the hub in the integration of several abiotic stimuli and JA-dependent defences (Inigo *et al.* 2012*b*). Other subunits of the Mediator complex are also involved in transduction signalling in response to a wide spectrum of environmental stress and developmental processes. *Med16*, known in *Arabidopsis* as *SFR6*, is implicated in both SA- and JA-mediated defence gene expression, and in tolerance to *Pseudomonas syringae* infection (Wathugala *et al.* 2012). *Med8* in *Arabidopsis* is also involved in regulation of pathogen resistance and acts both independently and in concert with *Med25. Med8* also regulates flowering time (Kidd *et al*. 2009), cell expansion and organ growth (Xu and Li 2012).

**Figure 3.**
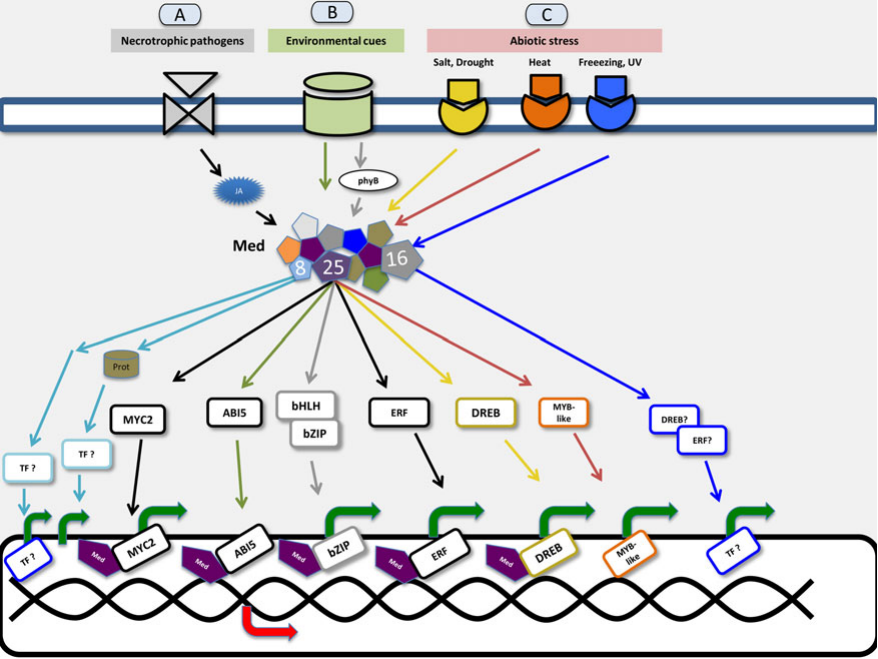
Repertoire of signalling pathways in plants in response to environmental cues, biotic and abiotic stresses, with Mediator as a central actor in the scene. (A) A JA-dependent signalling cascade activated in response to necrotrophic pathogens, implying Med25 in the Mediator complex, MYC2 TF and the activation of MYC2 target genes. (B) Two signalling cascades: the first activated in response to environmental cues through Med25 in the Mediator complex, ABI5 TF, leading to a repression of ABI5 target genes; and the second, phyB as receptor of light signals (shade), acting through Med25 in Mediator and bHLH and bZIP TFs and the expression of target genes. In a category of ‘activation by destruction’, from signals originating in environmental cues, Med25 is proteasome-degraded and coupled to the activation of *FLOWERING LOCUS T* (*FT*), florigen production and flowering in plants. Med8 involved in cell expansion and organ growth by a route independent of Med25. (C) Salt and drought stresses are sensed by a signalling cascade through Med25 in Mediator, DREB and MYB-like TFs and the expression of their respective genes. Under freezing or UV light stressing conditions, the Mediator subunit involved is Med16. The Mediator complex regulates transcription by mediating interactions between transcriptional activators and RNA PolII.

It is not fully understood how the multiprotein Mediator complex interprets and differentiates between specific, separated or simultaneous environmental cues. It is also not clear how the complex orchestrates the participation of specific subunits for integration of positive and negative regulatory information. In part, the integrative regulatory function is achieved by differentially specific interaction with a plethora of TFs (Fig. 3). The inferred interactome network for PFT1 (*Med25*, *At1g25540*) (BioGRID ID: 24378) using available data from *A. thaliana* with BioGRID version 3.2.99 available online (Stark *et al.* 2011) including physical and genetic data excluding self-interactions contains a total of 47 interactions at low confidence level. The outstanding interactors are of TF families (Fig. 2, Table 2).

**Table 2.** Transcription factors and proteins in interaction with PFT1 (Med25) in *A. thaliana* inferred from the BioGRID database.

Interactor	Gene ID	Short description
ERF1	821902	Ethylene-responsive transcription factor 1B
MYC2	840158	Transcription factor MYC2
AT4G39070	830062	B-box type zinc finger-containing protein
AT3G23220	821900	Ethylene-responsive transcription factor ERF095
AT4G18450	827576	Ethylene-responsive transcription factor ERF091
AT3G23230	821901	Ethylene-responsive transcription factor ERF098
DREB2A	830424	Dehydration-responsive element-binding protein 2A
AT4G17880	827511	Transcription factor MYC4
BZIP	843221	Basic leucine-zipper 8
ABI5	818199	Protein abscisic acid-Insensitive 5
AT4G34040	829550	RING/U-box domain-containing protein
ERF15	817680	Ethylene-responsive transcription factor 15
AT5G46760	834719	Transcription factor ATR2
ORA59	837125	Ethylene-responsive transcription factor ERF094
MYB104	817236	myb domain protein 104
WRKY10	842009	Putative WRKY transcription factor 10
AT2G15530	816045	RING/U-box domain-containing protein
RAP2.2	820643	Ethylene-responsive transcription factor RAP2-2
RRTF1	829591	Redox responsive transcription factor 1
		Ethylene-responsive transcription factor ERF109
AT5G29000	833026	myb family transcription factor
HRB1	834983	Protein dehydration-INDUCED 19-7

It has been demonstrated that interactions between transcriptional activators and Mediator subunits involve a two-step binding mechanism that induces conformational changes in the Mediator subunit–activator–DNA complex (Wands *et al.* 2011) or in the bimolecular complex of Mediator subunit–transcriptional regulator, with changes in the energetic and structural parameters of the involved proteins, changes that in turn modify their binding affinity (Blomberg *et al.* 2012). Recent mass spectrometry and dynamic transcriptome analysis indicates that 17 Mediator subunits in *Saccharomyces cerevisiae* during non-stress conditions are phosphorylated at multiple sites. Phosphorylation presumably prevents stress response gene transcription under non-stress conditions, supporting the idea that a dynamic and differential Mediator subunit phosphorylation contributes to gene regulation in eukaryotic cells (Miller *et al.* 2012). In addition, analysis of the effect of different phytohormones and stresses on the transcript level of Mediator subunit genes in *Arabidopsis* revealed that environmental cues impact the stoichiometric ratios of Mediator subunits by affecting differentially the transcription of the respective genes (Pasrija and Thakur 2012). Furthermore, alternative splicing may regulate the activity of some Mediator subunits: *Med12* and *Med19* are alternatively spliced in human endothelial cells (Rienzo *et al.* 2012).

## Master Regulators in Signalling Cascades in Response to Abiotic Stress

Plants have evolved a variety of elaborate mechanisms to respond and adapt to a broad range of environmental stressors. In abiotic stress, as in biotic stress, extensive gene networks finely regulate the molecular mechanisms that lead to the assembly of an integral stress response. These molecular networks are intercalated and hierarchical nodes mediate crosstalk. The master regulators sit at these nodes.

### The Mediator complex is also involved in the abiotic stress response

In abiotic stress, as in biotic stress, the Mediator complex plays a critical role. As noted previously, Mediator is an integrator of regulatory signals that converge on promoters of stress-responsive genes. In plants, several Mediator subunits have been functionally linked to gene transcription regulation in response to diverse stress-specific signalling pathways (Kim *et al*. 2006). For example, Med25 interacts with TFs in the pathways activated by salt (ZFHD1), drought (DREB2A) and heat stress (MYB-like protein); Med25 is also involved in regulating flowering time in response to light conditions (Rizhsky *et al*. 2004; Elfving *et al*. 2011). In germination of *Arabidopsis med25* mutants, the negative effect of salt stress is evident at a low concentration of NaCl (50 mM); these effects are even stronger than those due to a mutation in *dreb2a*, the gene encoding a TF involved in drought stress response (Fig. 3). It has been suggested that Med25 has the opposite function of DREB2A in the response to drought (Elfving *et al.* 2011). Med25 interacts with TFs through its conserved activator-interacting domain (amino acids 551–680); the kinetics of the Med25–TF interaction depends on the TF (Elfving *et al.* 2011). ZFHD1 is involved in response to drought and high salinity and is activated by ABA treatment. Overexpression of *ZFHD1* in *Arabidopsis* results in higher drought tolerance (Tran *et al.* 2007). DREB2A interacts with genes containing the dehydration-responsive element/C-repeat triggering gene expression due to cold or drought; in a constitutively active form, it enhances drought tolerance (Sakuma *et al.* 2006). MYB-like TFs, specifically MYB-At1g26580, were elevated when plants were exposed to a combination of drought and heat stress, indicating their possible participation in regulating the transcription of responsible genes to both stressors (Rizhsky *et al*. 2004).

Med16, another subunit of Mediator, was first described in *Arabidopsis* as *SENSITIVE TO FREEZING6* (*SFR6*), and was later identified as a component of the Mediator transcriptional co-activator. Med16 is implicated in cold- and drought-inducible gene transcription, in tolerance to freezing and osmotic stress, and the response to UV-C irradiation (Wathugala *et al.* 2012). Expression profile comparisons of Mediator subunits in rice and *Arabidopsis* show that 29 genes encoding Mediator subunits in rice and four in *Arabidopsis* are differentially expressed in at least one of three stress conditions (desiccation, cold or salt stress) (Mathur *et al.* 2011). *In silico* and genome-wide expression analysis of plant Mediator subunit genes under stress conditions revealed differential transcript abundance and alternative forms of Mediator complexes in different cell types and developmental stages (Mathur *et al.* 2011). The broad participation of the Mediator complex in different situations of abiotic and biotic stress is supported in part by the plasticity of the Mediator subunits to adopt alternative conformations that may enhance specific interactions among the subunits or with particular TFs (Fig. 3, Table 2).

### NPR1, another integrator of the response to abiotic stress

NPR1 is another master regulator of abiotic stress, although it perhaps does not have the same relevance to abiotic as to biotic stress. Rice plants engineered to overexpress *Arabidopsis NPR1* exhibit a lesion-mimic/cell death phenotype when exposed to a certain quality of light; rice plants overexpressing NPR1 are hypersensitive to light (Fitzgerald *et al.* 2004). *Arabidopsis* NPR1 acts as a negative regulator of the transcription of several rice genes: *rab21* (a rice dehydrin), *salT* (encoding salt-stress-induced protein) and *dip1* (encoding dehydration-stress-inducible protein). Transgenic rices expressing *Arabidopsis AtNPR1* are hypersensitive to salt and drought stresses (Quilis *et al.* 2008). AtNPR1 is likely a key component in the brassinosteroid-mediated increased tolerance to heat and salt stress (Divi *et al.* 2010). Brassinosteroids, a group of steroidal phytohormones, are implicated in regulation of plant cell growth and morphogenesis (Wang *et al*. 2012) as well as adaptation to biotic and abiotic stresses (Kutschera and Wang 2012).

### SnRK1, the SNF1-related kinases, play a role in the response to abiotic stress

The SNF1-related protein kinase 1 (SnRK1) in plants is homologous to sucrose-non-fermentation1 (SNF1) from yeast and AMP-activated protein kinase (AMPK) in mammals, and is a well-documented central regulator in pathways that signal energy deprivation in plants. Plants, unlike mammals and yeast, express a large family of SnRKs that are classified into three subgroups, SnRK1, SnRK2 and SnRK3, based on sequence similarities and domain structures. The SnRK1 subgroup, with only three members in *Arabidopsis*, is the most closely related of the subgroups to SNF1 from yeast and AMPK from animals (Hrabak *et al.* 2003). Energy deprivation results from abiotic stress associated with most environmental perturbations, such as oxygen hypoxia related to flooding, drought, extreme temperatures and even pathogen attack (Baena-Gonzalez 2010). KIN10 and KIN11, two representative kinases of the SnRK1 group, are involved in responses to darkness, hypoxia and herbicide (3-(3,4-dichlorophenyl)-1,1-dimethylurea) treatment in *Arabidopsis*. These kinases act through G-box binding TFs, specifically GBF5/bZIP2, which can bind the G-box *cis*-element present in the promoter of the dark-induced gene *DIN6*. Analysis of gene expression profiles under sugar and energy starvation conditions identified 278 genes co-activated by KIN10 and sugar starvation and co-repressed in sugar-treated seedlings (Baena-Gonzalez *et al.* 2007). These data place KIN10 and KIN11 as central integrators in the regulation of the transcription of genes involved in the response to energy starvation stress and in modulation of primary and secondary metabolism including protein synthesis. A recent study demonstrated that SnRK1-type kinases induce stress-responsive gene expression through translocation to the nucleus where it associates with target genes in response to oxygen deprivation under flooding conditions (Cho *et al.* 2012). Hormonal signalling in ABA, auxin and cytokinin pathways also exhibits connections with SnRK1. Abscisic acid is a central regulator of plant responses to osmotic stress (Hubbard *et al.* 2010). *Arabidopsis* plants overexpressing *SnRK1.1* have hypersensitivity to exogenous ABA (Jossier *et al.* 2009). Thus, the SnRK1 family members control hormone-mediated signalling in abiotic stress. The biological function of these central regulators may be amplified by their inherent kinase activity and their capacity to interact with and activate the transcription of specific target genes in the nuclear space.

Members of the SnRK2 subfamily are positive regulators (ABA dependent and independent) of responses to abiotic stresses such as water deficit, salinity, low temperature, and cadmium and oxidative stress (Kulik *et al.* 2011, 2012). Expression of each of the 10 members of the SnRK2 subfamily in rice (*Oryza sativa*) is activated by hyperosmotic stress; of these, three (*SAPK8*, *SAPK9* and *SAPK10*) are also induced by ABA. This indicates that the SnRK2 protein kinase family has evolved specifically for hyperosmotic stress signalling (Kobayashi *et al.* 2004). *Arabidopsis* *SRK2C* is an osmotic-stress-activated protein kinase. *Arabidopsis SRK2C*-knockout mutants exhibit drought hypersensitivity in their roots, whereas overexpressing *SRK2C* transgenic lines are drought tolerant. This improved drought tolerance results from up-regulation of stress-responsive genes *RD29A, COR15A* and *DREB1A*/*CBF3*. Interestingly, stress-responsive genes were not induced constitutively, suggesting the specific activity of SRK2C during stress (Umezawa *et al.* 2004). Similar results were observed when *TaSnRK2.4* from *Triticum aestivum* was overexpressed in *Arabidopsis* transgenic lines. *TaSnRK2.4* overexpression enhanced tolerance not only to drought, but also to salt and freezing stresses. Overexpression of the recombinant kinase caused no effect on growth when transgenic lines were grown under well-watered conditions, indicating that engineering plants to express *TaSnRK2.4* may improve performance during abiotic stress without impacting growth under normal conditions. The inferred interactome network for KIN10 (*At3g01090*) (BioGRID ID: 6592) using data available from *A. thaliana* with BioGRID version 3.2.99 available online (Stark *et al.* 2011) containing physical and genetic data excluding self-interactions contains a total of 23 interactions at low confidence level. The outstanding interactors are protein kinase family, proteasome-related proteins, TFs and unknown proteins (Fig. 2, Table 3). In the case of KIN11, the interactome network contains 47 and 18 total interactions with any confidence and low confidence level, respectively; this is similar to the KIN10 interactome, and furthermore includes phosphatase, sugar transporters and cyclic nucleotide-gated channels (Fig. 2, Table 4).

**Table 3.** Transcription factors and proteins in interaction with KIN10 (At3g01090) in *A. thaliana* inferred from the BioGRID database.

Interactor	Gene ID	Short description
FUS3	822293	B3 domain-containing transcription factor FUS3
SNF4	837423	Sucrose non-fermenting 4-like protein
AT5G36250	833622	Putative protein phosphatase 2C 74
SKP1	843928	S-phase kinase-associated protein 1
PAD1	824289	Proteasome subunit alpha type-7-A
AT4G16360	827331	SNF1-related protein kinase regulatory subunit beta-2
PRL1	827272	Protein pleiotropic regulatory locus 1
CPK1	843928	Calcium-dependent protein kinase 1
GDU2	828681	Glutamine dumper 2
PGLCT	831472	Plastidic glucose transporter 4
CNGC13	826427	Cyclic nucleotide-gated channel 13
AT1G08530	837375	Hypothetical protein
SNF4	852763	Activating gamma subunit of the AMP-activated Snf1p kinase complex (contains Snf1p and a Sip1p/Sip2p/Gal83p family member); activates glucose-repressed genes, represses glucose-induced genes; role in sporulation, and peroxisome biogenesis
FUS5	839241	COP9 signalosome complex subunit 7
UBQ3	831899	Polyubiquitin 3
AT1G22160	838821	Hypothetical protein
AT1G78020	844137	Hypothetical protein

**Table 4.** Transcription factors and proteins in interaction with KIN11 (At3g29160) in *A. thaliana* inferred from the BioGRID database.

Interactor	Gene ID	Short description
SNF4	837423	Sucrose non-fermenting 4-like protein
AT4G16360	827331	SNF1-related protein kinase regulatory subunit beta-2
SKP1	843928	S-phase kinase-associated protein 1
SEX4	824383	Dual specificity protein phosphatase (DsPTP1) family protein
PAD1	824289	Proteasome subunit alpha type-7-A
AT5G51910	835266	Transcription factor TCP19
PRL1	827272	Protein pleiotropic regulatory locus 1
CDKC	830891	Cyclin-dependent kinase C-1
GDU2	828681	Glutamine dumper 2
JAZ3	821055	Protein TIFY 6B
ZML2	841585	GATA transcription factor 28
SNF4	852763	Activating gamma subunit of the AMP-activated Snf1p kinase complex (contains Snf1p and a Sip1p/Sip2p/Gal83p family member); activates glucose-repressed genes, represses glucose-induced genes; role in sporulation and peroxisome biogenesis
AT4G25920	828698	Hypothetical protein
AT1G07310	837242	Calcium-dependent lipid-binding domain
GDU4	817013	Glutamine dumper 4
ATERDJ2A	844334	Translocation protein SEC63
AT1G19450	8838529	Sugar transporter ERD6-like 4
CNGC18	831339	Cyclic nucleotide-gated channel 18

These results indicate the importance of SNRK as a sensor and master regulator of the energetic and metabolic status of plant cells as well as active participation during adaptation to diverse abiotic stressors.

### Target of rapamycin, another master integrator in the response to abiotic stress

Target of rapamycin (TOR) is a serine/threonine kinase conserved in fungi, insects, mammals and photosynthetic eukaryotes. Target of rapamycin is a master regulator of cell growth and proliferation having a central role in regulation of cell growth and development (Kunz *et al.* 1993; Ren *et al.* 2011). Target of rapamycin integrates intracellular signals that depend on nutrient availability, cellular energy status (ATP) and extracellular signals such as growth factors. Also, TOR is another example of a molecular player that integrates signals originating under abiotic stress. Eukaryotic TORs are conserved proteins of ∼280 kDa that have 40–60 % sequence homology at the amino acid level. In yeast, isoforms *TOR1* and *TOR2* have with 80 % of amino acid similarity, a partially redundant function (Kunz *et al.* 1993), whereas in animals and plants there is a single copy of *TOR*. In *Arabidopsis*, loss-of-function mutants lead to embryonic lethality (Menand *et al.* 2002; Ren *et al.* 2011).

Rapamycin, an antiproliferative drug produced by *Streptomyces hygroscopicus* (Schmelzle and Hall 2000) originally described as an antifungal agent (Vezina *et al.* 1975), binds to FKBP12 and this complex inhibits TOR activity (Sormani *et al.* 2007). In early studies of plants, rapamycin insensitivity was explained in part due to the finding that none of the FKBP homologues in *Arabidopsis* were able to form a ternary complex with TOR in the presence of rapamycin (Mahfouz *et al.* 2006; Sormani *et al.* 2007). Although this result suggested that rapamycin does not affect TOR function in plants, in the unicellular green alga *Chlamydomonas reinhardtii* TOR and FKBP12 homologues have been identified and characterized, and *Chlamydomonas* cells are sensitive to rapamycin (Crespo *et al.* 2005). Recently, by analysis of site-specific phosphorylation of *Arabidopsis* S6Ks, a key substrate and mediator of TOR and a sensitive molecular and biochemical marker of endogenous TOR PK activity, it was found that rapamycin does effectively inhibit TOR protein kinase activation by glucose (Xiong and Sheen 2012). The two blocks of HEAT motifs at the N-terminus of TOR enable it to interact with Regulatory-Associated Protein of TOR (RAPTOR). Regulatory-Associated Protein of TOR in turn enlists TOR kinase substrates (Andrade and Bork 1995). A TOR-regulated pathway controls growth via regulation of translation through the TOR substrate ribosomal p70 S6 kinase (Dufner and Thomas 1999). In *Arabidopsis*, RAPTOR1 interacts with TOR (through the HEAT repeats) as well as S6K1, and the activity of S6K1 is affected by osmotic stress. Ectopic expressions of both *AtRAPTOR1* and *AtS6K1* in tobacco (*N. tabacum*) render the plant's osmotic stress insensitive; this indicates that the inhibition of S6K1 in plants under osmotic stress is under the control of TOR (Mahfouz *et al.* 2006). Similarly, TOR inactivation leads to a nutrient-starvation response, suggesting that TOR is involved in the response to nutrient deficiency (Barbet *et al.* 1996). Down-regulation of TOR by RNAi reduces organ growth and causes early senescence and transcriptomic and metabolomic perturbations, and sugar and amino acid accumulation. Moreover, plants overexpressing *TOR* accumulate more biomass and are more resistant to metabolic and osmotic stress (Dobrenel *et al.* 2011), and the level expression of *TOR* correlates inversely with the length of the primary root under salt concentrations. Conversely, constitutive expression of *TOR* alleviates the detrimental effect of osmotic stress (Deprost *et al.* 2007). Many more studies of *TOR* and its partners have been performed in other eukaryotes than in plants, but the work has burgeoned in recent years. The available data situate TOR kinase as a prominent link between environmental constraints and plant responses.

## Evolution of Master Regulators of Signalling Pathways in Response to Stress

As has been exemplified in the previous sections, master regulators in plants are key components in the processes of stress sensing, signal transduction, response signal integration, gene expression remodelling, energetic and metabolic status tuning, and modification of development and growth patterns. All these processes demand energy and generally have a metabolic cost and, therefore, are fully activated only when cells are under biotic or abiotic stress (Santos *et al.* 2011; Atkinson and Urwin 2012). Cells employ a wide variety of control checkpoints in order to regulate which defence mechanisms are activated in order to surpass the adverse conditions and which mechanisms are deactivated or remain down-regulated. Importantly, these regulatory molecules are responsible for rapid and efficient activation of defence mechanisms that will lead the plant to adaptation. Nearly every organism, from bacteria to multicellular eukaryotes, have sensory systems that allow measuring environmental cues; in other words, encoded in genotypes is the ability to produce distinct phenotypes determined by the variations in the environment (Pigliucci 2005). Thus it is rational that some of the stress-responsive regulatory networks and their master regulators are present in different organisms, and work under similar mechanisms as observed in plants to promote acclimation to the stressing conditions.

### AMPK/SNF1/SnRK1 protein kinases: master regulators of the energy status in eukaryotes

The SNF1s/SNF1-related kinases/AMPKs are evolutionarily conserved sensors and master regulators of the energetic and metabolic states of the cell. These conserved regulators are found in all eukaryotic organisms from simple unicellular fungi (yeast SNF1) to roundworms (AMP-activated kinase), insects (AMPK), plants (SnRK1) and animals (AMPK) and are the decisive regulators of the gene expression in response to energy or nutrient depletion-stressing conditions and, in some instances, are regulators of the activity of key metabolic enzymes (Polge and Thomas 2007). In general, these protein kinases function as heterotrimeric complexes that require a catalytic α-subunit and regulatory β- and γ-subunits for their structural stability and kinase activity. The number of complexes that can be formed varies significantly between organisms. For example, humans express several isoforms of each subunit that form AMPK: two α-subunits, two β-subunits and three isoforms of the γ-subunit. All variants are encoded by different genes; this diversity means that 12 different heterotrimeric complexes can be formed (Hardie 2011). *Saccharomyces cerevisiae* encodes one catalytic α-subunit (*Snf1*), three β-subunits (*Gal83, Sip1* and *Sip2*) and a single γ-subunit (*Snf4*) (Celenza *et al.* 1989). In plants, as previously described, SnRK kinases are grouped into three subfamilies: SnRK1, SnRK2 and SnRK3. The SnRK1 subfamily members have structural organization similar to AMPK and Snf1. The SnRK2 and SnRK3 subfamilies show some degree of sequence similarity to the catalytic α-subunits from yeast and mammals, but they do not functionally complement the yeast *snf1* deletion mutant (Hrabak *et al.* 2003). Despite this, the different subunits show remarkable evolutionary cross-species conservation at the sequence level. In the α-subunits, catalytic activity requires phosphorylation of a conserved threonine residue: Thr210 in SNF1, Thr172 in AMPK and Thr175 in SnRK1.1/KIN10 (Polge and Thomas 2007; Ghillebert *et al.* 2011). The amino acid sequences of the α-subunits from SNF1, AMPK and SnRK1 have 48 % identity overall, a percentage that rises to 60–65 % in the kinase domain. This noteworthy conservation among species indicates that an ancient kinase complex might have appeared 1.5 billion years ago, the estimated time when fungi, plants and mammalian kingdoms diverged. This also suggests that the complex originally evolved as a mechanism to regulate energy and carbon metabolism and response to starvation (Hardie 2007; Polge and Thomas 2007).

Interestingly, not only structural and regulatory aspects are shared among AMPK/SNF1/SnRK1 kinases but also the mechanism of enzyme activity and gene transcription control. Like the mechanism of SnRK activities in plants during stress discussed above, in mammals AMPK maintains cellular energy homoeostasis by regulating metabolic processes and responses to variable environments and energetic and metabolic stresses. AMP-activated protein kinase triggers catabolic pathways that produce ATP (Marsin *et al*. 2002; Tomas *et al*. 2002; van Oort *et al*. 2009; Wu and Wei 2012) and in parallel inhibits several anabolic processes via direct phosphorylation of key metabolic enzymes (Carling and Hardie 1989; Hardie and Pan 2002; Wakil and Abu-Elheiga 2009; Bultot *et al.* 2012), ensuring that general metabolism proceeds in accordance with nutrient availability and the cellular energy status (Hoppe *et al.* 2009). In addition to direct regulation of key metabolic enzymes, AMPK activates transcription of several genes involved in cellular adaptation to stress by modulating the activity of TFs (Li *et al*. 2011) and co-activators (Bungard *et al.* 2010; Hardie 2011; Mihaylova and Shaw 2011). The *S. cerevisiae* AMPK orthologue, SNF1 protein kinase, exerts very similar activities as a master regulator of the energy homoeostasis in yeast (Sanz 2003). SNF1 senses nutrient and energy starvation stress and through positive or negative regulation of gene expression and phosphorylation of TFs, and key metabolic enzymes activates metabolic processes to produce ATP coupled to inhibition of energy-expensive biosynthetic processes (Woods *et al.* 1994). SNF1 regulates the transcription of a large set of genes including those involved in the metabolism of alternative carbon sources, gluconeogenesis, respiration, transport and meiosis (Hedbacker and Carlson 2008). SNF1 catalytic activity also increases in response to a variety of stressors such as sodium ion stress, oxidative stress, alkaline pH, treatment with antimycin A (respiratory chain inhibitor) (Hong and Carlson 2007), nitrogen limitation (Orlova *et al.* 2006) and heat stress (Hahn and Thiele 2004). These regulatory roles emphasize the key participation of AMPK and SNF1 kinases in promoting protective actions and processes that confer maximal stress tolerance in eukaryotic life forms.

### The TOR system: master regulator of cell growth and proliferation in almost all eukaryotes

In contrast to SNF1/SnRK1/AMPK kinases that are activated by a decrement in the cellular energetic status, TOR kinase is activated by favourable and nutrient-rich conditions. The TOR signalling pathway transmits this information of wellness to the machinery of various energy-consuming processes such as mRNA translation, protein synthesis and cell proliferation.

In yeast and animals, there are two TOR complexes: TORC1, which contains three major proteins (TOR1 or TOR2, KOG1/RAPTOR and GbetaL/LST8) and TORC2, which is composed of TOR2, LST8/GbetaL and SIN1/RICTOR. These conserved components of the TORC1 complex are found in plants (Inoki and Guan 2006). Target of rapamycin is a vital protein, as inhibition of *TOR* expression results in early embryonic death in *Drosophila melanogaster* (Zhang *et al*. 2000), *Caenorhabditis elegans* (Long *et al.* 2002), mice (Gangloff *et al.* 2004) and *Arabidopsis* (Menand *et al.* 2002). In yeast and mammalian cells, TOR signalling regulates numerous biological processes including ribosomal biogenesis, protein translation, cell size regulation and cell proliferation (Chen *et al*. 2012*a*; Davie and Petersen 2012).

Since TOR controls cell growth by integrating nutrient and environmental information, it is reasonable that unfavourable growth conditions regulate the TOR activity. In yeast, TORC1 activity is down-regulated in response to carbon, nitrogen or phosphate starvation and in response to high salinity, high temperatures and oxidative stress (Loewith and Hall 2011). Studies in *Drosophila* and mammalian cells have shown that TOR signalling is inhibited under hypoxic stress. Hypoxia up-regulates the expression of *REDD1* and *REDD2* (Scylla and Charybdis in *Drosophila*), proteins that act downstream of Akt, an activator of TORC1 (Brugarolas *et al.* 2004; Reiling and Hafen 2004; Miyazaki and Esser 2009). DNA damage and redox stress also down-regulate TOR signalling (Feng *et al.* 2005; Sarbassov and Sabatini 2005; Feng 2010). Genotoxic stress inhibits Sestrin1 and Sestrin2 (transcriptional targets of the DNA damage sensor p53) and activates AMPK, thereby inhibiting TOR pathway activity (Budanov and Karin 2008). Inactivation of mammalian TOR (mTOR) by RNA interference in HeLa cell culture drastically reduces the synthesis of heat shock proteins, suggesting a key role for mTORC1 in transcriptional responses to proteotoxic stress (Chou *et al.* 2012). RNA-microarray analysis of the transcriptome of HEK293 cells (embryonic kidney cells) exposed to moderate hypertonicity showed that mTOR regulates the transcription of osmostress response genes, revealing a previously unappreciated role of mTOR in regulating transcriptional mechanisms that control gene expression during cellular stress responses in human cells (Ortells *et al.* 2012). In *A. thaliana* grown in the presence of nitrate excess, the overexpression of *AtTOR* causes an increment in the primary root in comparison with the control, relieving the inhibition caused by nitrogen excess. Similar results were observed in the primary roots of overexpressing plants under osmotic stress (Deprost *et al.* 2007).

The existence of TOR signalling pathways in all eukaryotic photosynthetic organisms, from unicellular green algae to animals and land plants, is now indisputable. A recent phylogenetic analysis of the TOR pathway revealed that the two TOR complexes and most TOR pathway components originated prior to the Last Eukaryotic Common Ancestor and that some accessory inputs were incorporated during evolution. These features reinforce the idea of van Dam *et al.* (2011) in relation to the fact that this is a vital pathway, highly conserved and flexible, capable of adapting to fulfil the changing needs of growth and development.

### The Mediator complex: a versatile, master regulator of transcription conserved from yeast to metazoans and plants

In higher eukaryotes, the tight regulation of the expression of several hundreds of genes is achieved through a variety of sequence-specific TFs. Differential engagement of the RNA PolII initiation machinery to gene promoters is crucial to control the transcription. Polymerase II is capable of relaxing and rewinding the DNA; however, PolII by itself is incapable of recognizing promoters and initiating transcription. For that a large pre-initiation complex (PIC) is required. The PIC is composed of more than 60 proteins including several general TFs. The large multisubunit Mediator complex is responsible for bridging diverse DNA-bound transcriptional regulators to the RNA PolII initiation machinery (Kornberg 2007). Mediator facilitates PolII recruitment and enhances the formation of the PIC by facilitating the assembly of an enhancer/core promoter loop complex containing activators, general TFs, PolII and cohesions. Mediator then enhances the phosphorylation of the C-terminal domain of PolII via the general TF TFIIH. The Mediator complex also regulates the release of hindered PolII, enhances the re-initiation and coordinates RNA capping, splicing and polyadenylation (Borggrefe and Yue 2011).

As a master integrator of regulatory signals, Mediator has a key role in the transcription of stress-responsive genes, and several subunits are responsible for activation of stress-specific signalling pathways in plants, fungi and mammals. The Srb11/Ssn8 cyclin subunit of the Mediator Kinase domain of human, plant and fungal pathogens (*Cryptococcus neoformans*, *Candida albicans*, *Fusarium verticillioides* and *Fusarium gramineaurum*) participates in the regulation of a general stress response by means of repressing nutrient responsive functions and transcription of genes related to the production of toxins and pigments, and controlling the cell wall integrity (Shim and Woloshuk 2001; Enjalbert *et al*. 2003; Zhou *et al*. 2010; Wang *et al*. 2011). Elimination of the Med32 subunit in *S. cerevisiae* and *Schizosaccharomyces pombe* results in phenotypes with increased sensitivity to oxidative and salt stress and ethanol (Linder *et al.* 2008; Koschubs *et al.* 2009). Elimination of the Med32 subunit in *C. albicans* causes the same stress-sensitive phenotypes, indicating conservative roles. Interestingly, the elimination of Med32 revealed the additional roles of this subunit in the transcription of virulence-related genes with significant impact on the *ALS* adhesins (Uwamahoro *et al.* 2012). These results indicate that the Mediator complex is a decisive player in virulence, filamentation and biofilm formation in fungus species.

The Mediator complex is generally known as a co-activator; however, the Mediator complex also acts as a negative regulator of transcription. Thus, the Mediator complex can act as a co-activator, co-repressor and general TF (Kornberg 2007). This set of features was decisive during the evolutionary diversification in eukaryotes. A recent comparative genomic analysis of Mediator subunits from 70 eukaryotes, including parasitic protists, diatoms, oomycetes, amoebae, green and red algae, land plants, fungi and animals, led to interesting conclusions on its evolutionary origin. The analysis showed that all yeast Mediator subunits have structural counterparts in insects and animals and allowed the identification of a set of core subunits that are traceable in most eukaryotic taxa. Interestingly, no Mediator subunit is specific to animals. All these data indicate the existence of an ancient four-module Mediator complex that appeared early in eukaryotic evolution (Bourbon 2008). The high interspecies conservation observed for these molecular subunits assembled in a dynamic complex (Mediator complex) involved in signalling pathways, with prominent regulatory functions of gene expression, noteworthy implied in response to stress, explains and supports sufficiently their early evolutionary appearance and preservation in eukaryotes.

## Conclusions

All organisms have mechanisms to repair damaged structures, scavenge toxic species and produce protective molecules (compatible solutes) and protein stabilizers (chaperones), among many other processes that alleviate alterations in cellular energy, metabolism and cell division. It is no surprise that the main signalling pathways that regulate cell growth, metabolism, senescence and apoptosis under normal conditions also regulate the stress responses. Harmonized regulation of the expression of the entire battery of genes related to stress responses and defence activities is successfully achieved commonly by the action of master regulators. The master regulators Mediator, NPR1, SnRK1 and TOR act as hubs to ensure that cellular resources are optimized. These factors play central regulatory roles in plant responses to biotic and abiotic stresses and are also important in signalling pathways active during normal growth. Possibly the best understood of these plant master regulators is Mediator, a protein complex in plants that is composed of at least 34 subunits that interact dynamically, offering in the organism a wide variety of options for the assortment of elements involved in the co-activation of gene transcription in response to biotic and abiotic stresses. This macromolecule assembly exemplifies how plants (and other organisms) have evolved to respond efficiently to changes in the environment by integrating several pathways through key master regulators.

Despite broad knowledge of the genes positively and negatively regulated by master regulators, the intricate networks of gene transcription regulation in response to stressors are still not completely understood. Integrative approaches are required in order to elucidate all the possible interactions among stress signals, master transcriptional regulators, TFs (activators and repressors), responsive genes and crosstalk between different stress signals. Systems biology approaches are proving to be of much help in the study and elucidation of complex regulatory networks as these approaches integrate theory, omics data and mathematical models. It is becoming possible to obtain an integrative image of the cellular status at different levels of organization: transcriptional, proteomic, metabolic and even interactomic. This high degree of integration allows a deeper understanding of complex processes and the reconstruction of networks with the possibility for characterization and quantification of the relationship of the genotype to the phenotype. Systems biology and omics approaches have been used to elucidate some key regulatory pathways and their components in plant responses to abiotic stress (Hirai *et al.* 2004; Cramer *et al.* 2011; Weckwerth 2011; Obata and Fernie 2012). For example, a recent data warehouse for maize called OPTIMA-DW (http://www.optimas-bioenergy.org/optimas_dw) has been created. This system biology project is a comprehensive compendium of transcriptomic, proteomic, metabolomic and ionomic analyses from maize grown under a large set of controlled stress conditions (drought, cold, nutrient deficit) or developmental stages (Colmsee *et al.* 2012).

Identification of participants in the different interactome-stress networks will help us to discover key regulatory targets susceptible to modification, opening the possibility for design of integral strategies for crop improvement. Genes targeted for modification must be stress-responsive elements and adjustment of expression must confer some degree of adaptation to adverse conditions. An important characteristic is that the activity of target genes must not result in an energetic or metabolic cost that will affect growth under non-stressing conditions. Modification of the activity of master regulators is a promising strategy for plant stress improvement that should increase the plasticity of the plant responses and adaptation as many genes and enzymes are under their control. However, to avoid adverse side-effects, such as metabolic burden, energy exhaustion or overall poor growth performance under non-stressing conditions, modification of master regulator activities must be carefully tuned. Another strategy is to modify the activity of general repressors that negatively modulate the transcription of a wide range of genes. This strategy will modify the expression of a specific set of genes that impact the response towards a stressor without the need to modifying all activities of master regulators. This strategy could also be applied to TFs associated with the signalling pathways controlled by master regulators to up-regulate the expression of certain genes.

It will also be important to elucidate the crosstalk between signalling pathways and identify master regulators that are active nodes during different stressing conditions. A genome-scale regulatory model of the *Arabidopsis* genome predicted that 10 TFs are the most influential regulatory hubs (these are *KAN3*, *AP2*, *ANAC036*, *KAN*, *AtTLP3*, *AGL46*, *MYB29*, *PHD finger*, *AETRF1/ERF1* and *MYB121*). Twelve gene subnetworks that have high clustering coefficients were identified, indicating high normalized indices of connectivity between the genes involved in the subnetwork. Interestingly, four of these subnetworks are involved in biotic and abiotic stresses (response to other organisms, response to heat, SAR, response to salt stress and immune response), suggesting that *A. thaliana* has evolved regulatory networks with high connectivity as a way to respond efficiently and dynamically to changing environments (Carrera *et al.* 2009). Future work will help in the identification and resolution of a core regulatory module in plants such as that already identified in yeast using a system-level analysis (Kim *et al*. 2012). Using the regulatory information from experimentally identified signalling and transcriptional networks in yeast, a global regulatory network was constructed with a stringent cutoff. From this, a core regulatory module was identified that interconnects different stress-responsive subregulatory networks. The core acts as an information processor on which all the environmental signals converge, and these signals are efficiently interpreted and common stress responses can be induced. This type of integrative study will increase our knowledge of how cells respond to numerous stressors with a minimum number of internal molecular components.

## Sources of Funding

This paper was made possible by the financial support from PROMEP-SEP-Redes Temáticas de Colaboración 2011-UAZ-CA-138 and by Fondo Institucional de Fomento Regional para el Desarrollo Científico, Tecnológico y de Innovación- Doctores 174509.

## Contributions by the Authors

V.E.B.-H. and S.F.-V. contributed equally to this paper.

## Conflict of Interest Statement

None declared.
